# Comparison of Guided Exercise and Self-Paced Exercise After Lumbar Spine Surgery: A Randomized Controlled Trial

**DOI:** 10.3390/life15071070

**Published:** 2025-07-04

**Authors:** Seong Son, Han Byeol Park, Kyeong Sik Kong, Byung Rhae Yoo, Woo Kyung Kim, Jae Ang Sim

**Affiliations:** 1 Department of Neurosurgery, Gil Medical Center, Gachon University College of Medicine, Incheon 21565, Republic of Korea; sonseong@gilhospital.com (S.S.);; 2 Department of Community Sports Science, Korea National Sport University, Seoul 05541, Republic of Korea; 3 Department of Orthopedic Surgery, Gil Medical Center, Gachon University College of Medicine, Incheon 21565, Republic of Korea

**Keywords:** back muscles, exercise, low back pain, lumbar spine, postoperative care, rehabilitation

## Abstract

Background: The efficacy of postoperative exercise rehabilitation after spine surgery is controversial, and a protocol for exercise treatment and detailed outcomes based on functional activity have not yet been established. This study aimed to determine the efficacy of exercise rehabilitation after lumbar spine surgery. Methods: A prospective, randomized controlled trial was conducted in 40 patients who underwent lumbar spine surgery (20 patients each in the exercise and control groups) for 12 weeks. Clinical outcomes were assessed using the visual analog scale (VAS) for pain and EuroQol-5 Dimensions 5-Level version (EQ-5D-5L). Body proportions, including body mass index, total muscle mass, and body fat percentage were analyzed. Functional activity was evaluated based on the range of motion of the lumbar spine, strength and endurance of lumbar flexion/extension, flexibility, 6 min walking test, single-leg stance, coordination, and gait pattern analysis. Results: The exercise group showed significantly greater improvement in VAS for pain (66.67% versus 20.00%, *p* < 0.001) and EQ-5D-5L (45.56% versus 20.00, *p* = 0.039) compared to the control group. Serial assessment revealed significant improvement in strength of lumbar flexion/extension, 6 min walking test, single-leg stance, coordination, and gait patterns in the exercise group compared to the control group. In particular, the single-leg stance time for the affected leg improved more markedly in the exercise group (280.9% versus 48.7%, *p* < 0.001). Conclusion: Tailored postoperative exercise after lumbar spine surgery is effective in reducing pain and enhancing functional recovery, including strength and balance.

## 1. Introduction

Postoperative exercise rehabilitation is widely recognized as one of the most effective and popular modalities for postoperative care [1]. Following lumbar spine surgery, various adjuvant treatments are employed to enhance the outcomes, including medication, massage, cognitive behavioral training, electrical nerve stimulation, and exercise [2,3,4]. Among these strategies, exercise rehabilitation, encompassing core and functional exercises, has been extensively used in clinical settings to strengthen the lumbar paraspinal muscles and improve postoperative pain [5,6,7,8]. Additionally, recent meta-analyses have underscored the clinical significance of exercise treatment for low back pain (LBP) compared to other conservative approaches [9,10,11,12].

Exercise rehabilitation after lumbar spine surgery can take many forms, including supervised physical therapy sessions, home exercise programs, and group exercise classes. Each modality has its advantages and challenges. For instance, supervised physical therapy ensures proper technique and progression but can be costly and time-consuming for patients [13]. Home exercise programs offer flexibility and convenience but may suffer from lower adherence and a lack of supervision [13]. Group exercise classes can provide social support and motivation but may not be tailored to individual needs [14]. The choice of exercise modality can significantly impact patient outcomes, including pain reduction, functional improvement, and overall quality of life [15].

Although the clinical efficacy of postoperative exercise in reducing pain and disability is generally supported, some controversy remains regarding the optimal types and intensities of exercises, the timing of initiation, and the appropriate duration of the rehabilitation program [16,17,18,19,20]. The growing evidence supports the effectiveness of structured and individualized exercise programs in enhancing postoperative recovery [21,22]. For instance, core-strengthening exercises that focus on spinal stabilization and posture correction have been shown to alleviate pain and improve function [23]. However, the lack of standardization contributes to confusion among surgeons and clinicians about specific exercise regimens, outcome tracking, and coordinated rehabilitation planning following lumbar spine surgery [24]. The continued absence of objective, standardized protocols for individualized training and assessment remains a persistent challenge.

We hypothesized that systematic individualized exercise rehabilitation following lumbar spine surgery would lead to significant improvements in pain and functional activity. To investigate this hypothesis, we developed an exercise treatment protocol tailored to patients’ baseline functional activity levels. This protocol was applied to patients who had undergone lumbar spine surgery, and their clinical progress and functional outcomes were systematically tracked. The primary endpoint was clinical outcome, including pain reduction and quality-of-life improvement. The secondary endpoint focused on functional activity, assessed through various exercise-related parameters.

## 2. Materials and Methods

### 2.1. Trial Design

This prospective, single-center, randomized, open-label trial enrolled patients who underwent surgery for lumbar degenerative diseases (see in Supplementary Materials).

### 2.2. Ethics

The entire research process was conducted in accordance with the 1964 Helsinki Declaration and its subsequent amendments. Approval for the study was obtained from the Institutional Review Board of our institute on 14 February 2022 (GDIRB2022-095), and the study was registered with the Clinical Research Information Service (CRIS) under registration number KCT0009533.

### 2.3. Aims

This study aimed to explore the superiority of postoperative exercise rehabilitation over a control group in improving the clinical course and the functional activity of patients after lumbar spine surgery.

The primary endpoint of the study was to analyze the improvement in clinical outcome, including degree of pain and quality of life during a 12-week treatment period. The secondary endpoint was quantitative change in functional activities’ outcome during the follow-up period.

### 2.4. Sample Size

The sample size was calculated as follows:

$$N=\frac{{2(Z_{1-\alpha /2}+Z_{1-\beta })}^{2}\;\delta^{2}}{{(\mu }_{1}{-\mu_{2})}^{2}}$$

Based on the results of previous clinical trials of postoperative exercise [1,25], the average difference in the effect value after treatment (*μ*_1_ − *μ*_2_) was set to 1.5, and the standard deviation of the main effect value (*δ*) was set to 1.2. Using the above formula [26], with a significance level of 5% and a power of 95%, the sample size was calculated to be 16 patients in each group. Considering a dropout rate of 20%, a total of 20 patients were recruited for each group.

### 2.5. Participant Recruitment

Participants were recruited from the inpatient clinics of two neurosurgeons specializing in spine surgery. Each potential subject was screened to determine eligibility based on the inclusion/exclusion criteria.

The inclusion criteria were as follows: (1) adults aged 20–70 years who had undergone single-level lumbar spine surgery (from L1 to S1); (2) at least 6 weeks had passed postoperatively following discectomy, decompression, or fusion for lumbar degenerative disease, including lumbar disc herniation, spinal stenosis, or spondylolisthesis; (3) patients who could ambulate sufficiently to perform postoperative exercise treatment; and (4) patients who voluntarily decided to participate in the trial and follow the trial protocol.

The exclusion criteria were as follows: (1) those who could not perform postoperative exercise, such as those with paralysis (motor grade ≤ 3), bladder or bowel dysfunction, concurrent lower-extremity disease, or extreme pain, making daily activities impossible; (2) patients who were unable to understand consent or had difficulty performing the exercise protocol independently (e.g., patients with mental disorders or reduced cognitive function); (3) pregnant women or women who planned to become pregnant during the study period; and (4) those ineligible to participate in this trial based on the researcher’s judgment.

### 2.6. Time Frame

This study was conducted over one year, from February 2023 to January 2024, with a follow-up period of 12 weeks. Detailed explanations, informed consent, and baseline characteristics were obtained prior to registration. Patients were randomly assigned to either the exercise group or control group. However, due to the nature of the face-to-face exercise program, blinding of participants and investigators was not feasible.

**Figure 1:**
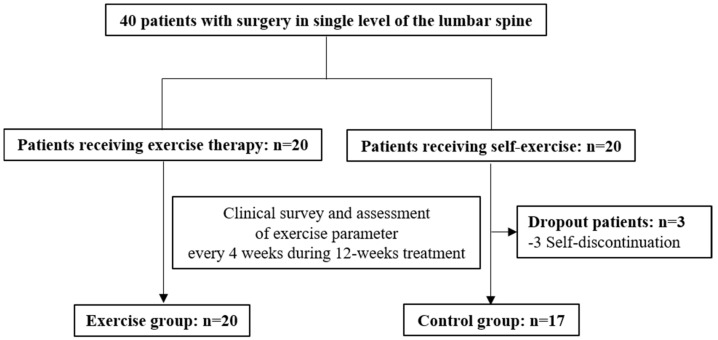
Flow diagram illustrating patient recruitment and study process.

The exercise group received instructions to visit the exercise room twice a week for 12 weeks to perform rehabilitation exercise according to tailored protocols provided by a certificated physical therapist. The control group was instructed to perform self-exercise routines based on conventional printed postoperative exercise guidelines, which comprised similar exercise movements to those of the exercise group. Regular visits for clinical surveys, measurement of functional activity, and treatment monitoring were scheduled at pretreatment and at 4, 8, and 12 weeks after treatment initiation (Figure 1).

### 2.7. Grading and Individualized Tailored Exercise

Prior to the start of the study, baseline data on the exercise capabilities of 411 patients who had undergone single-level lumbar spine surgery at least 6 weeks earlier were collected. The parameters of exercise capacity included the following: (1) the range of motion of the lumbar spine (flexion and extension); (2) trunk strength measured by the strength of flexion and extension; (3) trunk muscular endurance assessed by the endurance of flexion and extension; (4) flexibility of the whole body posterior chain measured using a forward flexion measuring device; (5) cardiopulmonary capability evaluated through the 6 min walking test; (6) general balance assessed by the single-leg stance test; (7) coordination ability, measured by the timed up-and-go test; and (8) gait balance analysis based on single support time of the normal or affected leg.

A grading system was developed to categorize each patient’s exercise ability into five stages. An individualized exercise program was then designed for each participant, targeting a level one to two grades higher than their baseline. For example, if a patient was classified at strength grade II, the intervention aimed to train them toward grade III or IV. If the patient’s strength grade improved after four weeks, the training goal was subsequently adjusted to match the newly assessed grade.

### 2.8. Protocol of Postoperative Exercise and Control Group

Baseline exercise capacity was assessed for each patient upon enrollment, after which the patients were randomly assigned to one of two groups. The exercise group underwent exercise sessions with a physical therapist at an affordable intensity based on baseline exercise capacity, lasting for 1 h twice a week (24 times over 12 weeks) following an initial education session. The control group received a 1 h, one-on-one education session on the same exercise program and were instructed to perform home training based on their self-awareness of intensity. This educational program was customized on an individual basis to help patients recognize their current statuses and understand how to address their individual problems.

The exercise treatment administered to both groups consisted of the following: (1) 10-min warm-up comprising static and dynamic stretching of the hip flexor, hip adductor, gluteus, hamstring, quadriceps, and calf muscles; (2) 3–5 sets (15 repetitions per set, with 30 s of rest) of various core strength training exercises, including pelvic tilt, bridge, curl-up, bird-dog, dead-bug, hip abduction, hip extension, flank, side flank, calf raise, lunge, squat, step up, seated row, and lat pulldown, with a gradual increase in exercise loads [27]; (3) single-leg stance exercises with increasing difficulty, such as visual occlusion and unstable surfaces [28]; (4) coordination exercise involving simple repetitive up-and-go exercise using both upper and lower extremities (participants were instructed to sit in a chair with armrests, stand up following the audio command “start,” turn at a distance of 3 m, and then sit back on the chair; measurements were repeated 3 times, and the average value was recorded) [28]; and (5) cardiorespiratory endurance exercise, consisting of 10–15 min of walking on a treadmill with gradual increase in exercise loads based on pretreatment grading and self-feedback during the cool down period [29].

### 2.9. Outcome Assessment

During the study period, screening, exercise training and education, clinical surveys, and data collection were carried out by two certified physical therapists and one investigator. Baseline data included age, sex, clinical diagnosis, pathological level, surgery, medical history, affected side, and interval from surgery to exercise initiation. Additional data were collected from the baseline laboratory tests.

The body proportion analysis, including body mass index, total muscle mass, and total body fat percentage using Inbody^®^ (Inbody, Seoul, Republic of Korea), were conducted at pretreatment and at 4, 8, and 12 weeks after treatment initiation in both groups.

The clinical outcome parameters comprised the visual analog scale (VAS) of LBP, ranging from 0 to 10 points, and the EuroQol 5-Dimension 5-Level version (EQ-5D-5L) [30]. These clinical outcomes were assessed at pretreatment and at 4, 8, and 12 weeks after treatment initiation in both groups.

The functional activities’ outcome was assessed using various exercise parameters. All exercise parameters described were assessed at pretreatment and at 4, 8, and 12 weeks after treatment in both groups. The range of motion of the lumbar spine was defined as the mean of two recordings of the maximal degree of flexion and extension measured in the straight standing position using the digital goniometer HALO^©^ (HALO Medical Devices, Sydney, Australia) [31]. Trunk strength was defined as the average of three measurements of strength using a digital handheld dynamometer (Micro FET2^®^, Seed technology, Bucheon, Republic of Korea) on the sternal notch during full flexion or on the C7-T1 spinous process during full extension for 3 s in the sitting position [32]. Trunk muscle endurance was evaluated once to maintain 50% of each subject’s maximum flexion and extension strength in the sitting position [33]. Lumbar flexibility was confirmed as the average of two measurements of the points of both fingertips when the upper body was bent for 3 s in a position on the floor with the legs stretched forward [34]. Cardiopulmonary capability was assessed based on the distance covered when quickly walking for 6 min [35]. General balance was evaluated by averaging three measurements of the maximum time required to stand on one foot [36]. Coordination capability was defined as the average of two measurements of time spent to stand from sitting in a chair, walk 6 m, and return to the chair [36]. Gait pattern was defined as the average of three measurements of the percent (%) of unilateral foot support time during the 6 min gait test using the Neurogait^®^ app (Salted, Hanam, Republic of Korea) [37,38].

### 2.10. Adjuvant Treatment

All patients were permitted to continue taking their prescribed medications at their discretion. Decisions regarding maintenance, tapering, or cessation of medication were made based on each patient’s and clinician’s preference during the treatment period. Patients were also given the option to continue or discontinue their usual self-exercises prior to study according to their preferences. However, invasive treatments that could substantially impact clinical outcomes, such as lumbar intervention or acupuncture, were not allowed.

### 2.11. Statistical Analysis

Data management and statistical analyses were conducted using SPSS (version 27.0; IBM Corporation, Armonk, NY, USA). Pearson’s chi-square test, Fisher’s exact test, the independent *t*-test, Mann–Whitney U test, and Kruskal–Wallis test were employed to analyze collected data.

Values are presented as means ± standard deviations (SDs) or as medians and interquartile range (IQRs), depending on the distribution of the data. Statistical significance was defined as *p* < 0.05.

## 3. Results

### 3.1. Baseline Characteristics

Of the 40 participants initially registered, 37 were included in the final cohort (20 in the exercise group and 17 in the control group) after exclusion owing to loss to follow-up and simple consent withdrawal. The 37 study subjects consisted of 12 men and 25 women with an average age of 62.68 ± 12.34 years. Baseline characteristics, including age, sex, clinical diagnosis, pathological level, surgery, medical history, affected leg, and interval from surgery to the initiation of exercise treatment, did not differ between the two groups (Table 1).

In addition, the baseline laboratory test results did not differ between the two groups (Table 2).

### 3.2. Change in Body Proportion

Overall, body proportions, including body mass index, total muscle mass, and total body fat percentage, did not differ between the two groups from initiation to final follow-up (Table 3), and no difference was identified between the two groups in the degree of change in the final values, compared with that of the initial values (Table 3).

### 3.3. Clinical Outcome

**Figure 2:**
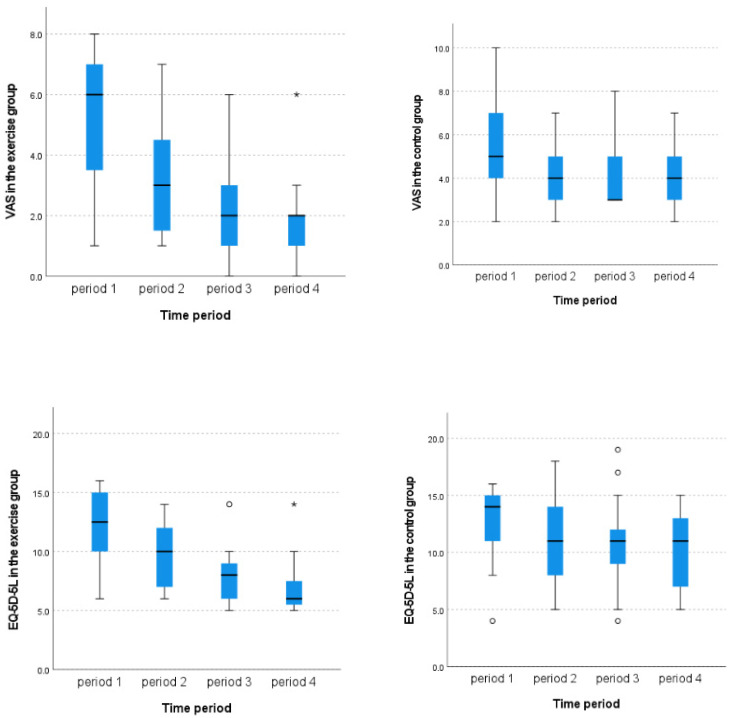
Longitudinal analysis depicting the visual analog scale (VAS) for pain and EuroQol-5 Dimensions 5-Level version (EQ-5D-5L) for quality of life in both groups. * indicates a statistically significant difference compared to the previous period (* *p* < 0.05).

Longitudinal analysis revealed that the VAS score for pain gradually decreased after 12 weeks in the exercise group (from 6.0 [IQR 3.25–7.0] to 2.0 [IQR, 1.0–2.0], *p* < 0.001, effect size = 0.299, non-parametric Kruskal–Wallis test), whereas the VAS score for pain did not show significant improvement during the study period in the control group (from 5.0 [IQR, 4.0–7.0] to 4.0 [IQR, 3.0–5.0], *p* = 0.212, non-parametric Kruskal–Wallis test). Similarly, the EQ-5D-5L score improved after 12 weeks in the exercise group (from 12.5 [IQR, 10.0–15.0] to 6.0 [IQR, 5.25–7.75], *p* < 0.001, effect size = 0.370, non-parametric Kruskal–Wallis test), whereas the EQ-5D-5L score did not show significant improvement during the study period in the control group (from 14.0 [IQR, 10.0–15.0] to 13.0 [IQR, 11.0–14.2], *p* = 0.147, non-parametric Kruskal–Wallis test) (Figure 2).

According to the comparative study between the two groups, the VAS score for pain and EQ-5D-5L score were significantly different between the two groups at 8 and 12 weeks after treatment initiation. Consequently, the degree of improvement in the VAS score for pain (66.67% [IQR, 57.14–86.46] versus 20.00% [IQR, 0.00–53.57], *p* < 0.001, effect size = 0.596, Mann–Whitney U test) and in the EQ-5D-5L score (45.56% [IQR, 31.43–53.85] versus 20.00% [IQR, 6.46–43.75], *p* = 0.039, effect size = 0.339, Mann–Whitney U test) was greater in the exercise group than in the control group (Table 4).

### 3.4. Functional Activities’ Outcome

Longitudinal serial analysis revealed that the ROM flexion, ROM extension, flexion strength, and extension strength significantly increased after 12 weeks in both groups (ROM flexion 77.0% increase [*p* < 0.001, non-parametric Kruskal–Wallis test], ROM extension 57.8% increase [*p* < 0.001, non-parametric Kruskal–Wallis test], flexion strength 20.8% increase [*p* = 0.002, non-parametric Kruskal–Wallis test], and extension strength 42.3% increase [*p* < 0.001, non-parametric Kruskal–Wallis test] in the exercise group; ROM extension 64.7% increase [*p* = 0.001, non-parametric Kruskal–Wallis test], ROM extension 43.9% increase [*p* = 0.024, non-parametric Kruskal–Wallis test], flexion strength 39.1% increase [*p* = 0.031, non-parametric Kruskal–Wallis test], and extension strength 47.4% increase [*p* = 0.013, non-parametric Kruskal–Wallis test] in the control group). However, flexion endurance, extension endurance, 6 min walking, single stance of affected side, coordination, and single-leg stance were improved significantly only in the exercise group (flexion endurance 53.4% improvement [*p* = 0.003, non-parametric Kruskal–Wallis test], extension endurance 8.7% improvement [*p* = 0.039, non-parametric Kruskal–Wallis test], 6 min walking 34.6% improvement [*p* < 0.001, non-parametric Kruskal–Wallis test], single stance of affected side 280.9% improvement [*p* = 0.028, non-parametric Kruskal–Wallis test], coordination 21.1% improvement [*p* < 0.001, non-parametric Kruskal–Wallis test], and gait pattern of affected side 16.2% improvement [*p* < 0.001, non-parametric Kruskal–Wallis test]). In contrast, the control group did not show significant improvement in above parameters (flexion endurance 0% improvement [*p* = 0.263, non-parametric Kruskal–Wallis test], extension endurance 6.1% improvement [*p* = 0.221, non-parametric Kruskal–Wallis test], 6 min walking 24.3% improvement [*p* = 0.272, non-parametric Kruskal–Wallis test], single stance of affected side 48.7% improvement [*p* = 0.834, non-parametric Kruskal–Wallis test], coordination 15.7% improvement [*p* = 0.134, non-parametric Kruskal–Wallis test], and gait pattern of affected side 10.1% improvement [*p* = 0.172, non-parametric Kruskal–Wallis test]). On the other hand, flexibility, single-leg stance of normal side, and gait pattern of normal side did not show significant improvement in either group (Table 5).

Comparative analysis between two groups reveled that the overall degree of improvement in most exercise parameters did not significantly differ between the two groups, except for the single-leg stance on the affected side, which improved by 280.9% (IQR, 23.7–326.7) in the exercise group compared to 48.7% (IQR, −22.0–254.0) in the control group (*p* < 0.001, effect size = 0.536, Mann–Whitney U test) (Table 5).

### 3.5. Side Effects

No side effects were noted, except for two cases of transient aggravation of LBP in the first 4 weeks in the exercise group. However, in these cases, the pain improved after continued treatment.

## 4. Discussion

Regarding clinical outcomes assessed by the VAS and EQ-5D-5L scores, the exercise group exhibited significantly greater improvement compared to the control group. These findings are consistent with previous systematic reviews supporting the efficacy of postoperative exercise [39,40]. The observed improvement in the VAS (66.67%) and EQ-5D-5L (45.56%) scores in the exercise group support prior research indicating that core stabilization exercises enhance paraspinal muscle activity and mass, thereby alleviating pain and improving quality of life [41,42,43,44,45]. Furthermore, the degree of VAS improvement in this study was comparable to or even exceeds that reported in several previous randomized controlled trials [8,16,46,47,48]. While comparisons are drawn indirectly, the magnitude of pain reduction and quality-of-life improvement represents a noteworthy accomplishment.

Baseline characteristics, including body composition, did not differ significantly between the two groups. Most functional activities’ outcome also showed no significant intergroup differences, with the notable exception of single-leg balance on the affected side, which improved more markedly in the exercise group. This difference likely stems from the intensive affected-leg exercise in the exercise group compared to self-training at home in the control group. However, serial analyses using the Kruskal–Wallis test revealed significant improvements in the exercise group in flexion endurance, extension endurance, 6 min walking distance, single-leg stance on affected side, coordination, and gait pattern on affected side. These improvements are likely attributable to the structured and progressive nature of the individualized exercise protocol in the exercise group, in contrast to the self-directed home exercises in the control group.

The individualized tailored exercise program demonstrated both safety and efficacy, with no serious adverse events reported. Only minor and transient worsening of LBP was observed. Unlike pharmacologic or procedural interventions, which carry risks of adverse events [49,50], this exercise-based approach proved to be a safe rehabilitation modality. Importantly, the exercise group showed high adherence and no dropouts during the 12-week intervention, likely reflecting patient satisfaction with face-to-face instruction and ongoing feedback from certified physical therapists.

This study had several limitations. First, due to the nature of the intervention, blinding of participants and investigators was not feasible, introducing potential bias. Second, we were unable to control for all confounding variables such as concurrent medication use or additional self-administered exercises. However, we endeavored to mitigate bias by allowing patients to maintain pre-study routines while disallowing new interventions during the study period. Third, the sample size was small and predominantly female, and the follow-up period was relatively short. Additionally, we did not assess the sensitivity or specificity of the clinical tests used to evaluate the main outcomes. Although these tests are widely used in clinical practice, their responsiveness in this specific patient population was not validated, which may limit the interpretability of our findings. Future studies should aim to validate the accuracy and responsiveness of these outcome measures in the context of lumbar spine rehabilitation.

Despite the introduction of various postoperative exercise programs, the lack of a standardized, evidence-based protocol remains a challenge [51]. Many existing exercise regimens rely on empirical methods without clearly defined goals or measurable outcomes [9]. Even advanced exercise treatments, such as patient-customized motor skill training programs in functional activities, remain unstandardized and overly complex for independent patient implementation [52,53]. To address these challenges, we have developed a grading system for exercise capability through the analysis of the postoperative population. This allowed us to categorize patients into five stages and deliver appropriately scaled, individualized exercise programs, adjusting intensity based on performance and compliance.

Despite the noted limitations, our study provides objective evidence supporting the efficacy and safety of tailored postoperative exercise programs based on baseline physical function. These findings are valuable as they incorporate detailed functional and clinical outcome measures. However, further research involving larger, more diverse populations and longer follow-up periods is warranted to confirm the long-term benefits of individualized rehabilitation after lumbar spine surgery.

## 5. Conclusions

This study highlights the efficacy and safety of individualized postoperative exercise treatment for patients following lumbar spine surgery. Tailored postoperative exercises have the potential to improve patient satisfaction and yield favorable outcomes. While further large-scale studies are needed to validate and generalize these findings, our results provide a foundation for the implementation of individualized postoperative exercise as a structured component of spine surgery rehabilitation.

## Figures and Tables

**Table 1:** Demographic data.

		Exercise Group (*n* = 20)	Control Group (*n* = 17)	*p* Value
Sex, male/female		7/13	5/12	0.717 ^a^
Age		59.50 ± 13.65	66.41 ± 9.69	0.090 ^b^
Diagnosis				0.213
	Disc herniation	13	9	
	Spinal stenosis	7	6	
	Spondylolisthesis	0	2	
Level				0.612 ^a^
	L1–L4	3	3	
	L4–S1	17	14	
Surgery				0.132 ^a^
	Discectomy	13	9	
	Laminectomy	7	5	
	Fusion	0	3	
Medical history				
	Diabetes	5	8	0.161 ^a^
	Hypertension	8	9	0.431 ^a^
	Others	2	1	0.562 ^c^
Affected side of leg, right/left		11:9	7:10	0.402 ^a^
Alcohol, yes/no		9/11	4/13	0.300 ^c^
Smoking, yes/no		6/14	1/16	0.097
Interval from surgery to treatment initiation (days)		17.0 (IQR, 13.5–29.0)	12.0 (IQR, 6.0–28.0)	0.125 ^d^

^a^. Pearson’s chi-square test; ^b^. independent *t*-test; ^c^. Fisher’s exact test; ^d^. Mann–Whitney U test; IQR, interquartile range.

**Table 2:** Baseline laboratory test.

	Exercise Group (*n* = 20)	Control Group (*n* = 17)	*p* Value
Serum glucose (mg/dL)	107.15 ± 14.57	128.14 ± 44.29	0.108 ^a^
High density lipoprotein cholesterol (mg/dL)	51.95 ± 14.24	50.78 ± 10.65	0.808 ^a^
Total cholesterol (mg/dL)	167.75 ± 24.02	154.82 ± 31.81	0.256 ^a^
Triglyceride (mg/dL)	146.55 ± 105.86	150.67 ± 139.62	0.939 ^a^
Blood urea nitrogen (mg/dL)	14.55 ± 7.39	16.15 ± 7.30	0.520 ^a^
Creatinine (mg/dL)	0.85 ± 0.46	0.079 ± 0.34	0.658 ^a^
Aspartate aminotransferase (U/L)	26.20 ± 10.73	21.69 ± 6.94	0.137 ^a^
Alanine aminotransferase (U/L)	21.60 ± 11.15	21.19 ± 11.47	0.914 ^a^
Gamma-glutamyltranspeptidase (U/L)	31.10 ± 22.19	27.88 ± 25.39	0.692 ^a^
Hemoglobin (g/dL)	12.37 ± 1.29	11.51 ± 1.55	0.110 ^a^
Hematocrit (%)	37.02 ± 4.02	34.75 ± 4.64	0.133 ^a^
White blood cell (/mm^3^)	7027.50 ± 2926.94	6088.80 ± 1441.43	0.219 ^a^
Platelet (/mm^3^)	247.85 ± 49.50	293.00 ± 110.19	0.123 ^a^
High-sensitivity C-reactive protein (mg/dL)	0.21 (IQR, 0.06–10.50)	0.05 (IQR, 0.05–0.06)	0.651 ^b^
Erythrocyte sedimentation rate (mm/h)	5.50 (IQR 2.00–10.50)	4.00 (IQR, 2.00–6.00)	0.277 ^b^

^a^. independent *t*-test; ^b^. Mann–Whitney U test.

**Table 3:** Change in body proportion.

		Exercise Group (*n* = 20)	Control Group (*n* = 17)	*p* Value
Height (cm)		161.91 ± 7.54	160.18 ± 8.67	0.525
Weight (kg)				
	Baseline	64.09 ± 10.81	65.24 ± 10.04	0.743
	4 weeks	64.19 ± 10.49	65.35 ± 9.58	0.730
	8 weeks	64.31 ± 10.61	64.85 ± 10.95	0.888
	12 weeks	64.46 ± 10.90	65.54 ± 10.38	0.762
	Change (%)	0.62 ± 2.32	1.26 ± 4.43	0.616
Body mass index (kg/m^2^)				
	Baseline	24.35 ± 2.64	25.49 ± 331	0.272
	4 weeks	24.39 ± 2.57	26.55 ± 3.22	0.249
	8 weeks	24.42 ± 2.63	24.91 ± 3.53	0.674
	12 weeks	24.48 ± 2.75	25.48 ± 3.47	0.354
	Change (%)	0.62 ± 2.33	1.26 ± 4.43	0.616
Total muscle mass (kg)				
	Baseline	24.02 ± 5.26	24.11 ± 4.19	0.951
	4 weeks	23.95 ± 5.03	24.26 ± 4.08	0.838
	8 weeks	24.11 ± 5.02	24.47 ± 4.69	0.834
	12 weeks	24.19 ± 5.07	24.33 ± 4.59	0.934
	Change (%)	0.74 ± 2.38	0.91 ± 5.06	0.461
Total body fat (kg)				
	Baseline	19.75 ± 4.88	20.26 ± 8.03	0.828
	4 weeks	19.98 ± 5.07	20.19 ± 7.16	0.919
	8 weeks	19.90 ± 4.82	19.53 ± 7.05	0.870
	12 weeks	19.96 ± 4.95	20.47 ± 6.20	0.791
	Change (%)	1.06 ± 7.94	1.03 ± 6.56	0.145
Total body fat percentage (%)				
	Baseline	30.39 ± 6.73	30.38 ± 9.48	0.997
	4 weeks	31.14 ± 6.46	30.46 ± 8.66	0.796
	8 weeks	30.94 ± 5.89	29.74 ± 8.29	0655
	12 weeks	30.96 ± 5.81	31.02 ± 7.32	0.979
	Change (%)	3.10 ± 11.51	7.66 ± 14.23	0.330

independent *t*-test.

**Table 4:** Clinical survey.

		Exercise Group (*n* = 20)	Control Group (*n* = 17)	*p* Value
VAS				
	Baseline	6.0 (IQR 3.25–7.0)	5.0 (IQR 4.0–7.0)	0.916
	4 weeks	3.0 (IQR 1.25–4.75)	4.0 (IQR 3.0–5.5)	0.125
	8 weeks	2.0 (IQR 1.0–3.0)	3.0 (IQR 3.0–6.0)	0.005
	12 weeks	2.0 (IQR 1.0–2.0)	4.0 (IQR 3.0–5.0)	<0.001
	Improvement (%)	66.67 (IQR, 57.14–86.46)	20.00 (IQR, 0.00–53.57)	<0.001
EQ-5D-5L				
	Baseline	12.5 (IQR, 10.0–15.0)	14.0 (IQR, 10.0–15.0)	0.598
	4 weeks	10.0 (IQR, 7.0–12.0)	11.0 (IQR, 8.0–14.0)	0.220
	8 weeks	8.0 (IQR, 6.0–9.0)	11.0 (IQR, 9.0–12.5)	0.004
	12 weeks	6.0 (IQR, 5.25–7.75)	11.0 (IQR, 6.5–13.0)	0.017
	Improvement (%)	45.56 (IQR, 31.43–53.85)	20.00 (IQR, 6.46–43.75)	0.039

Mann–Whitney U test; EQ-5D-5L, EuroQol-5 Dimensions 5-Level version; VAS, visual analog scale.

**Table 5:** Functional activities’ outcome based on exercise parameters.

		Exercise Group (*n* = 20)	Control Group (*n* = 17)	*p* Value *
ROM flexion (°)				
	Baseline	43.0 (IQR, 28.0–54.0)	30.5(IQR, 26. –36.0)	0.104
	4 weeks	58.0 (IQR, 49.0–65.0)	40.0 (IQR, 33.0–50.5)	0.002
	8 weeks	65.0 (IQR, 54.0–77.0)	43.5 (IQR, 38.2–52.5)	0.002
	12 weeks	80.0 (IQR, 60.0–85.0)	51.0 (IQR, 43.0–68.7)	<0.001
	Change (%)	77.0 (IQR, 32.2–163.6)	64.7 (IQR, 19.4–132.1)	0.369
	*p* value ^†^	<0.001	0.001	
ROM extension (°)				
	Baseline	19.0 (IQR, 15.0–23.0)	16.0 (IQR, 11.0–20.7)	0.341
	4 weeks	22.0 (IQR, 18.0–26.0)	20.5 (IQR, 10.75–25.5)	0.442
	8 weeks	26.0 (IQR, 20.0–30.0)	20.5 (IQR, 15.7–24.0)	0.200
	12 weeks	29.0 (IQR, 26.0–32.0)	22.5 (IQR, 17.2–25.5)	<0.001
	Change (%)	57.8 (IQR, 26.0–111.1)	43.9 (IQR, 17.2–88.6)	0.178
	*p* value ^†^	<0.001	0.024	
Flexion strength (kg)				
	Baseline	13.9 (IQR, 12.2–15.8)	13.0 (IQR, 9.65–16.8)	0.537
	4 weeks	16.4 (IQR, 13.5–19.0)	15.9 (IQR, 13.9–17.2)	0.407
	8 weeks	17.1 (IQR, 14.2–21.4)	16.65 (IQR, 16.0–20.2)	0.305
	12 weeks	17.5 (IQR, 15.1–21.9)	17.5 (IQR, 15.0–20.6)	0.168
	Change (%)	20.8 (IQR, 9.5–49.5)	39.1 (IQR, 9.1–74.9)	0.912
	*p* value ^†^	0.002	0.031	
Extension strength (kg)				
	Baseline	15.1 (IQR, 13.8–20.85)	16.5 (IQR, 12.35–20.65)	0.619
	4 weeks	19.5 (IQR, 17.5–22.6)	19.8 (IQR, 14.4–22.7)	0311
	8 weeks	22.2 (IQR, 18.2–25.0)	22.75 (IQR, 20.8–25.4)	0.626
	12 weeks	23.2 (IQR, 19.8–24.3)	22.9 (IQR, 19.8–27.0)	0.626
	Change (%)	42.3 (IQR, 6.5–68.2)	47.4 (IQR, 17.3–71.0)	0.741
	*p* value ^†^	<0.001	0.013	
Flexion endurance (second)				
	Baseline	78.2 (IQR, 18.4–120.0)	90.0 (IQR, 37.1–120.0)	0.298
	4 weeks	120.0 (IQR, 65.0–120.0)	62.0 (IQR, 28.1–120.0)	0.987
	8 weeks	120.0 (IQR, 66.0–120.0)	120.0 (IQR, 56.8–120.0)	0.912
	12 weeks	120.0 (IQR, 90.0–120.0)	120.0 (IQR, 93.1–120.0)	1.000
	Change (%)	53.4 (IQR, 0–140.0)	0.0 (IQR, 0–147.3)	0.169
	*p* value ^†^	0.003	0.263	
Extension endurance (second)				
	Baseline	110.4 (IQR, 43.0–120.0)	90.5 (IQR, 28.7–120.0)	0.987
	4 weeks	120.0 (IQR, 64.0–120.0)	97.1 (IQR, 25.9–120.0)	0.730
	8 weeks	120.0 (IQR, 94.0–120.0)	120.0 (IQR, 71.8–120.0)	0.838
	12 weeks	120.0 (IQR, 100.0–120.0)	120.0 (IQR, 81.3–120.0)	0.962
	Change (%)	8.7 (IQR, 0–79.34)	6.1 (IQR, 0–219.6)	0.743
	*p* value ^†^	0.039	0.221	
Flexibility (cm)				
	Baseline	0.8 (IQR, −17.0–8.6)	−4.7 (IQR, −11.0–1.15)	0.464
	4 weeks	1.0 (IQR, −6.6–10.5)	0.25 (IQR, −6.10–5.60)	0.369
	8 weeks	2.3 (IQR, −1.9–14.4)	−1.1 (IQR, −4.7–7.0)	0.305
	12 weeks	7.1 (IQR, 0.1–10.2)	2.3 (IQR, −3.8–9.2)	0.320
	Change (cm)	7.75 (IQR, 0.13–17.60)	5.70 (IQR, 1.35–10.95)	0.537
	*p* value ^†^	0.233	0.089	
6-min walking (m)				
	Baseline	420.0 (IQR, 390.0–455.0)	376.5 (IQR, 240.0–413.2)	0.028
	4 weeks	480.0 (IQR, 450.0–533.0)	409.5 (IQR, 312.5–501.1)	0.009
	8 weeks	513.0 (IQR, 486.0–548.0)	403.5 (IQR, 337.5–496.8)	0.002
	12 weeks	553.0 (IQR, 506.0–620.0)	388.8(IQR, 337.5–520.9)	<0.001
	Change (%)	34.6 (IQR, 23.4–46.1)	24.3 (IQR, −9.4–61.4)	0.542
	*p* value ^†^	<0.001	0.272	
Single stance_normal side (second)				
	Baseline	28.1 (IQR, 5.8–47.5)	4.0 (IQR, 1.67–19.18)	0.002
	4 weeks	63.0 (IQR, 8.68–120.0)	7.4 (IQR, 3.81–20.65)	0.008
	8 weeks	80.0 (IQR, 713.0–120.0)	5.3 (IQR, 3.07–25.53)	<0.001
	12 weeks	58.9 (IQR, 22.4–120.0)	7.9 (IQR, 2.56–21.92)	0.002
	Change (%)	128.8 (IQR, 23.7–326.7)	62.2 (IQR, −22.0–254.0)	0.400
	*p* value ^†^	0.214	0.280	
Single stance_affected side (second)				
	Baseline	9.76 (IQR, 2.82–63.8)	3.61 (IQR, 1.66–23.66)	0.028
	4 weeks	62.00 (IQR, 4.32–112.0)	4.02 (IQR, 1.71–13.34)	<0.001
	8 weeks	70.00 (IQR, 8.29–100.79)	4.71 (IQR, 2.42–30.06)	<0.001
	12 weeks	70.00 (IQR, 7.20–120.0)	4.74 (IQR, 2.72–8.03)	<0.001
	Change (%)	280.9 (IQR, 23.7–326.7)	48.7 (IQR, −22.0–254.0)	<0.001
	*p* value ^†^	0.028	0.834	
Coordination (second)				
	Baseline	7.44 (IQR, 6.60–9.12)	8.42 (IQR, 6.53–15.25)	0.017
	4 weeks	6.52 (IQR, 6.16–7.33)	7.47 (IQR, 6.54–11.79)	0.012
	8 weeks	6.11 (IQR, 5.50–6.39)	7.38 (IQR, 6.32–11.63)	<0.001
	12 weeks	5.94 (IQR, 5.42–6.26)	7.09 (IQR, 6.12–10.06)	<0.001
	Change (%)	−21.1 (IQR, −31.1–−7.2)	−15.7 (IQR, −48.8–4.0)	0.888
	*p* value ^†^	<0.001	0.134	
Gait pattern_normal side (% of gait cycle)				
	Baseline	40.0 (IQR, 36.0–46.0)	39.0 (IQR, 36.0–43.5)	0.472
	4 weeks	41.0 (IQR, 40.0–44.0)	38.5 (IQR, 35.0–39.7)	0.254
	8 weeks	41.0 (IQR, 39.0–43.0)	40.0 (IQR, 38.5–42.0)	0.723
	12 weeks	42.0 (IQR, 41.0–43.0)	40.0 (IQR, 38.25–43.0)	0.179
	Change (%)	2.38 (IQR, −7.50–11.11)	2.54 (IQR, −4.76–16.01)	0.826
	*p* value ^†^	0.731	0.453	
Gait pattern_affected side (% of gait cycle)				
	Baseline	36.0 (IQR, 32.0–37.0)	35.5 (IQR, 33.5–37.0)	0.791
	4 weeks	37.0 (IQR, 36.0–40.0)	38.0 (IQR, 35.5–39.7)	0.623
	8 weeks	39.0 (IQR, 36.0–41.0)	37.0 (IQR, 36.0–38.7)	0.950
	12 weeks	41.0 (IQR, 40.0–42.0)	39.0 (IQR, 36.2–40.7)	0.190
	Change (%)	16.2 (IQR, 5.2–25.0)	10.1 (IQR, 1.3–18.4)	0.535
	*p* value ^†^	0.001	0.172	

IQR, interquartile range; * Mann–Whitney U test; ^†^ non-parametric Kruskal–Wallis test.

## Data Availability

The data presented in this study are available on request.

## Supplementary Materials

The following supporting information can be downloaded at: https://www.mdpi.com/article/10.3390/life15071070/s1, Figure S1: CONSORT 2010 flow diagram; Table S1: CONSORT 2010 checklist.
