# Supplementary figures and images for: Comparison of Guided Exercise and Self-Paced Exercise After Lumbar Spine Surgery: A Randomized Controlled Trial
Source: Life (Basel). 2025 Jul 4;15(7):1070. doi: 10.3390/life15071070 (PMC12298251; doi:10.3390/life15071070)

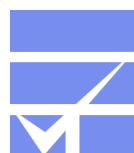

## CONSORT 2010 Flow Diagram

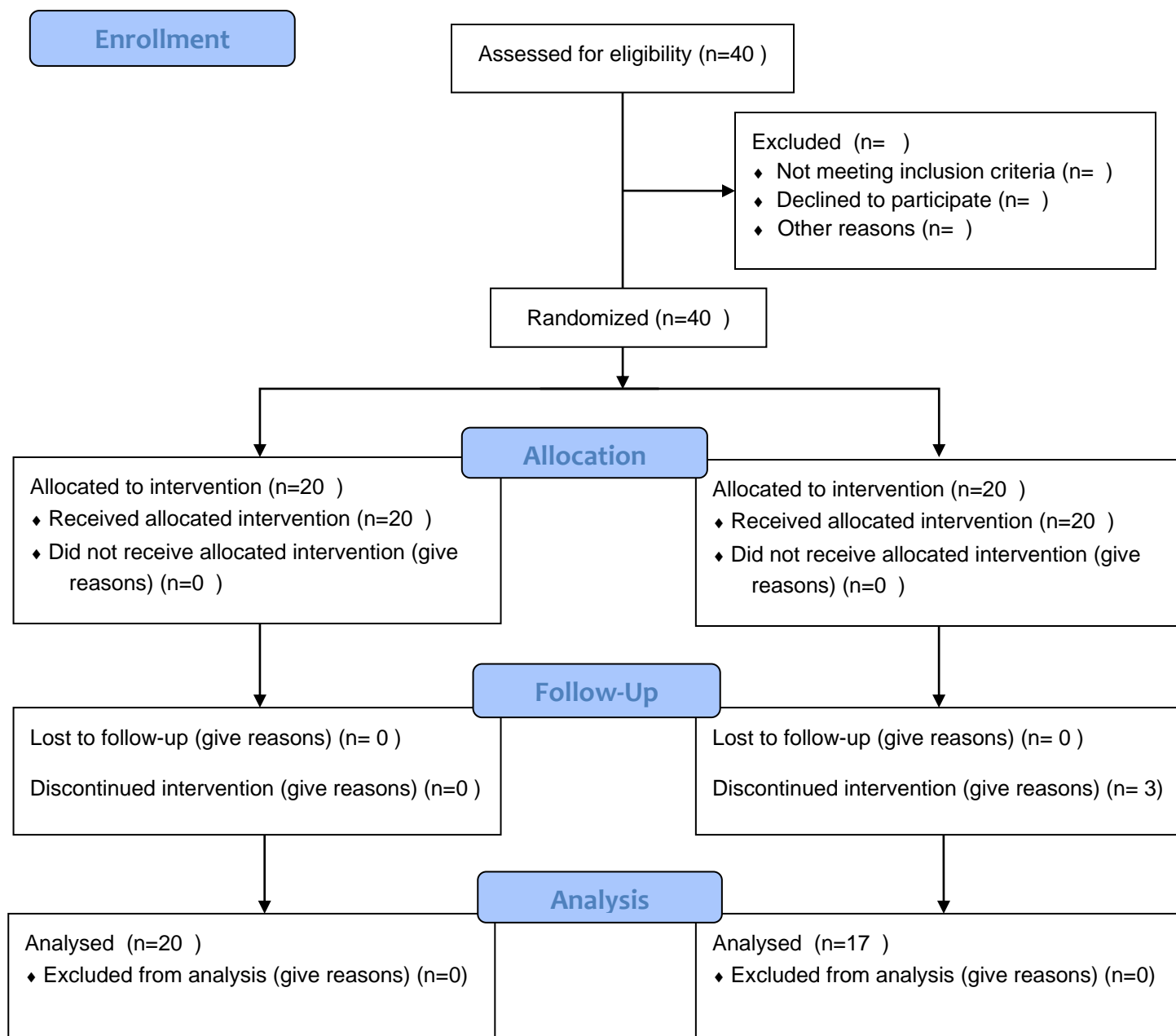

Supplement: Supplementary file 1 [file life-15-01070-s001.zip › Figure S1. consort-2010-flow-diagram.pdf]
